# Streamlining volumetric multi-channel image cytometry using hue-saturation-brightness-based surface creation

**DOI:** 10.1038/s42003-018-0139-y

**Published:** 2018-09-06

**Authors:** Yingrou Tan, Jackson Liang Yao Li, Chi Ching Goh, Bernett Teck Kwong Lee, Immanuel Weng Han Kwok, Wei Jie Ng, Maximilien Evrard, Michael Poidinger, Hong Liang Tey, Lai Guan Ng

**Affiliations:** 1 Singapore Immunology Network (SIgN), A*STAR (Agency for Science, Technology and Research), Biopolis, 138648 Singapore, Singapore; 2 National University of Singapore, 21 Lower Kent Ridge Rd, Singapore, Singapore 119077; 3 National Skin Centre, 1 Mandalay Road, 308205 Singapore, Singapore

## Abstract

Image cytometry is the process of converting image data to flow cytometry-style plots, and it usually requires computer-aided surface creation to extract out statistics for cells or structures. One way of dealing with structures stained with multiple markers in three-dimensional images, is carrying out multiple rounds of channel co-localization and image masking before surface creation, which is cumbersome and laborious. We propose the application of the hue-saturation-brightness color space to streamline this process, which produces complete surfaces, and allows the user to have a global view of the data before flexibly defining cell subsets. Spectral compensation can also be performed after surface creation to accurately resolve different signals. We demonstrate the utility of this workflow in static and dynamic imaging datasets of a needlestick injury on the mouse ear, and we believe this scalable and intuitive approach will improve the ease of performing histocytometry on biological samples.

## Abstract (web summary)

Yingrou Tan et al. present a method streamlining surface creation in 3D imaging by applying the hue-saturation-brightness transformed channels simultaneously. They show the utility of this approach by imaging ear skin following needlestick injury, observing immune cell infiltration.

## Introduction

A perennial problem when dealing with biological images is in translating qualitative data within the image into quantitative data for further analysis. Images contain invaluable information, such as spatial localization and cell-cell interactions, which other techniques such as immunophenotyping using flow cytometry and transcriptomics cannot easily provide. The field of image cytometry thus emerged to provide quantitative readouts for images of single cells to whole organisms^1,2^. Programs^3^ and algorithms for image segmentation to determine which objects certain pixels belong to (e.g., nuclei, cell cytoplasm), measurement of cellular or subcellular features after segmentation and machine-learning algorithms for classifying large datasets^4^ are indispensable tools that have been developed for carrying out image cytometry.

At present, a common way of quantifying three-dimensional data from image stacks is by creating virtual polygon mesh models, called surfaces, that simulate the shape of structures of interest (e.g., cells), typically by using commercial image processing software. Critical statistics such as the levels of marker expression, cell size and morphology can then be derived from these surfaces, which can be further analyzed using statistical analysis software, or converted and visualized in flow cytometry-style dot plots using software such as FlowJo in a method termed histocytometry^5^.

In recent times, the availability of a wider variety of fluorophores with distinct fluorescent spectra has led to the increasingly common use of multiplex staining^6^. This technique of multiplex staining coupled with histocytometry becomes a powerful tool to identify the tissue localization of different cell subsets^7–9^. However, a thorny issue faced by users when attempting histocytometry is how to deal with cells that are stained with more than one color, as they exist in multiple channels, when the process of surface creation is currently designed to work only on single channels. One of the existing approaches (which we term traditional) is to identify co-localizing pixels in order to generate new channels that can be used for surface creation^5,7^, while deleting these pixels from the original channels in order to prevent surface duplication (i.e., masking). However, this solution for generating surfaces becomes a bottleneck when dealing with cell types that express more than two markers, since co-localization channels are generated on a pairwise basis, and multiple co-localization channels will be needed to fully capture the possible combinations. Consequently, this process becomes exponentially laborious with the increasing number of markers. Furthermore, in this traditional workflow, the cycles of co-localization channel generation and masking is sequential, which means that early mistakes are directly carried over to subsequent work. Correcting them typically involves the wholesale repetition of previous work, which can be extremely time-consuming. In addition, since decisions on pixel co-localizations have to be made in advance before the complete set of surfaces are created, the results are often highly arbitrary and subjective, and mistakes easily remain undiscovered.

In this report, we demonstrate a way of circumventing limitations in surface creation by applying the Hue-Saturation-Brightness (HSB) surface creation workflow to deal with multi-parametric fluorescent images through mathematically transforming images into hue, saturation and brightness channels. Surfaces can then be created in a straightforward manner using the brightness channel. Color spaces provide a rational way of rendering color, and the most commonly known color space is the red–green–blue (RGB) space, where the individual displayed hues are represented by the differential combinations of the three primary colors. While fluorescence microscopy data are typically acquired in greyscale, the RGB space is usually used to represent multi-channel images in overlay. As the RGB color space is not intuitive in practical use, the HSB space was devised in the 1970s as a representation of color that is closer to human perception. HSB space is a cylindrical coordinate representation of the RGB space, and is commonly used for color selection in image editing or graphics applications. Unlike the RGB space that stores pixel data as separate intensities of the three primary colors, the HSB color space is rendered in three parameters—hue, which represents the base color; saturation, which represents the relative amount of white in a color (e.g., pink is essentially red with high white levels); and brightness, which represents the intensity of the signal^10^. The major advantage that HSB has over RGB space is that hue is independent of the signal intensity; whereas, the same information is distributed over each of the RGB channels.

We propose here the application of the HSB color space to the process of creating three-dimensional surfaces in multiplex stained microscopy images, and we further show here that the hue-saturation-brightness-based workflow can be used to simplify the process of surface creation (https://github.com/HSBsurfacecreation/Hue-Saturation-Brightness-based-surface-creation). The notable strengths of this approach are the flexibility it provides, tolerance to mistakes, generation of more complete surfaces, possibility of native hue color rendering, and its application to both static and dynamic images for quantitative analysis in 2-, 3-, or 4-dimensional manner. Using a needlestick injury of the mouse ear, we demonstrate how HSB surface creation can be applied for analyzing different cell populations—neutrophils, monocytes, and monocyte-derived cells as well as dendritic cells and macrophages in whole-mount immunostaining; and for differentiating tracks of dendritic cells and neutrophils in dynamic imaging. In addition, we illustrate how the biologist who may not be well versed in image analysis can carry out histocytometry for their images.

## Results

### Hue-saturation-brightness surface creation workflow

**Fig. 1:**
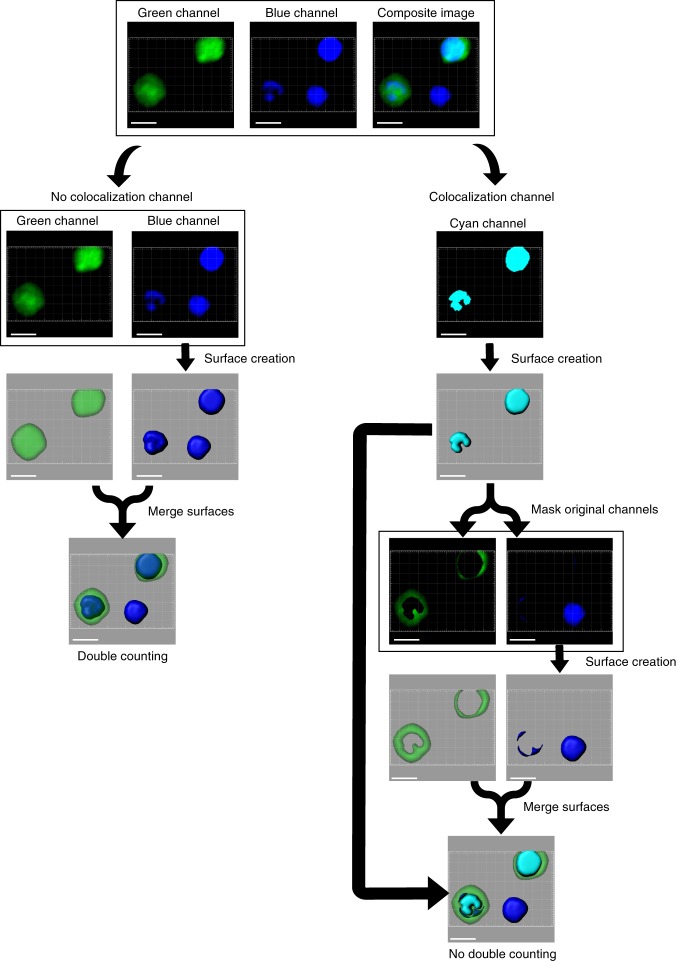
Illustration of masking using the co-localization channel. Surface creation using individual channels for a two-color image consisting of green and blue channels results in two surfaces being created for each double-positive cyan cell as it exists in both channels, thus the wrong number of surfaces are created. In order to achieve the right number of surfaces, a co-localization channel for double-positive cyan cells is built. The channel is then used to create three-dimensional surfaces, which is applied to the individual green and blue channels to remove the signal belonging to the cyan cell. This leads to the isolation of only single-positive cells in the masked channels. However, partial surfaces may be generated due to incomplete co-localization. Scale bar, 5 µm

**Fig. 2:**
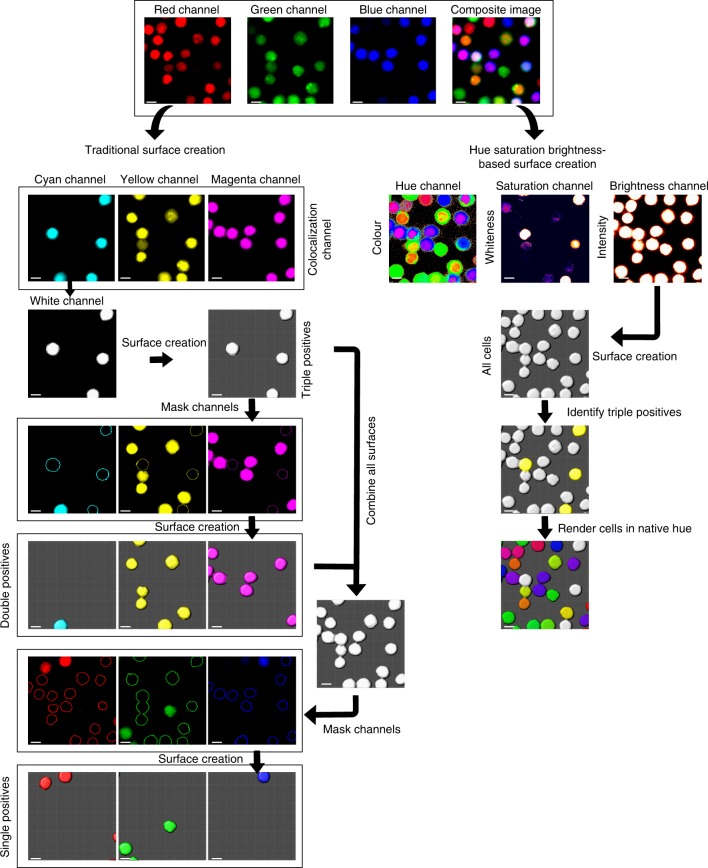
Comparison of traditional surface creation with hue-saturation-brightness surface creation. Traditional surface creation: For a three color image acquired in the red, green, and channels, it is necessary to first identify the different possible combinations of triple positive and double-positive cells using the co-localization channel for each combination. Cell surfaces of the triple and double-positive cells are created using image analysis software, surfaces are merged and used to mask the relevant cells in the original red, green, and blue channels to generate single-positive cells. Each of the individual channels is then used for surface creation and statistics extraction. Hue-saturation brightness surface creation: Hue, saturation, and brightness channels will be generated from the original image using the HSB algorithm. Any cell that expresses a marker will be captured in the brightness channel, thus making it possible to generate cell surfaces for all the cells in one channel. Triple positive cells can be identified using the saturation channel, and information from the hue channel can then be used to render the surfaces in their native hue. Scale bar, 5 µm

The traditional method of surface creation is cumbersome when dealing with multiplex stained samples, while the HSB surface creation workflow makes this process straightforward. This is because the traditional method relies on the tedious process of building co-localization channels to define subsets of cells stained with multiple markers to avoid duplicate surfaces (Fig. 1). Since co-localization channels are built sequentially in a pairwise manner, the number of channels required to define all possible combinations increase exponentially with the number of markers used (Supplementary Table 1). For example, a total of 63 channels are required for identifying cells stained with only 6 markers. We illustrate this problem with a relatively simple example using a three marker staining of cells on a glass slide (Fig. 2). Four different co-localization channels are needed to define all the double-positive and triple-positive cells. The cell surfaces created using the co-localization channels are then used to mask the signals from the original three channels to obtain the single-positive cells. The seven different channels are then individually used for surface creation to define all the different possible populations. In actual practice, this process is difficult, since more complex co-localization channels must be built from simpler channels, with no mistakes allowed along the process. The large number of steps required means that surface creation becomes a convoluted and tedious procedure with increasingly high risks of error.

In contrast, the brightness channel in the HSB surface creation workflow, which incorporates the intensity from all the channels simultaneously, can be directly used for surface creation, greatly streamlining the whole process (Fig. 2). In our application of the HSB color space, hue represents the contributions of the different markers to the color of the cell. Brightness represents the overall intensity, and any cell that is positive for any of the markers will be rendered in this channel. Finally, saturation represents how closely the three markers match in terms of relative intensity. For example, a bright green cell with background levels of red and blue signals has high brightness and low saturation, but a cell with equally weak red, green and blue signals has low brightness, but high saturation.

Information from the hue and saturation channels can be used to identify the different cell subsets. Each hue is given a numerical value and the spectrum is represented on a numerical linear scale (Supplementary Fig. 1a, Supplementary Note 1), independent of its brightness (Supplementary Fig. 1b). The hue value assigned to each surface thus enables the user to identify the specific combination of markers present on the cell. As the hue rendered is representative of only the two predominant colors, it is not possible to use the hue value to identify triple-positive cells. Identification of triple-positive cells is thus dependent on the saturation channel, which measures how closely the three colors match in individual channel intensities (Supplementary Fig. 1c). In the RGB space, pure white (or gray) is the combined presence of equal red, green and blue intensities. In the HSB color space, these uncolored hues are conventionally assigned the saturation value of 0, as is black (i.e., background), while pure colored hues are assigned the maximum possible value (e.g., 1.0 or 255). However, to make it easy to identify the triple-positive cells (i.e., white), we have inverted the saturation scale in the HSB surface creation workflow, such that cells with a strong predominant color (i.e., single- or double-positive) gets assigned a low saturation value, but pure black still always remains zero. This allows white cells to show-up strongly against a black background. It is then dependent on the user to identify which value to set as a threshold to define cells as being triple-positive (higher saturation signifies higher likelihood). As this decision is made only after the surfaces have already been created, it is a relatively trivial matter to change the threshold on the whim of the user. Also, for visualization purposes, it is possible to render the cell surfaces with a hue matching their native appearance, even before defining any population subsets, whereas this was impossible in the traditional workflow. This feature is particularly important for ascertaining the accuracy of cell segmentation, as the user can easily compare the hue of the cell surface with the actual data by eye. Proper cell segmentation is essential to the accuracy of the statistics extracted for downstream analysis.

### Application of HSB surface creation in a two-dimensional image

**Fig. 3:**
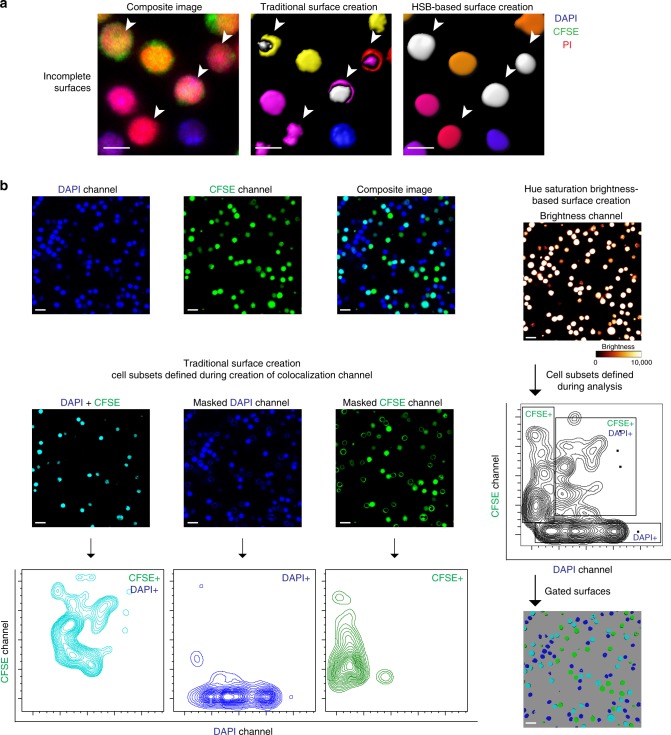
Surface quality and surface identity differs using traditional surface creation and HSB surface creation workflows. **a** Incomplete surfaces are generated with traditional surface creation, while the HSB surface creation workflow generates complete surfaces. Splenocytes stained with different combinations of DAPI, CFSE, and propidium iodide were mixed and imaged. The composite image of all three channels was compared with surfaces generated using the traditional surface creation and the HSB surface creation workflow. Surfaces from traditional surface creation are rendered as a composite of the different subsets, while surfaces from the HSB surface creation workflow are rendered in the median hue with triple-positive cells colored as white. Traditional surface creation results in a cell with surfaces belonging to two different subsets as the three markers do not fully overlap, while HSB surface creation results in a complete surface. Scale bar, 10 µm. **b** Cell subsets are defined early in the traditional surface creation workflow, but are only defined during the analysis step using the HSB surface creation workflow. Splenocytes stained with different combinations of DAPI and CFSE were mixed and imaged. Using the traditional surface creation workflow, the DAPI^+^ CFSE^+^ cell subset is defined during the creation of the co-localization channel. The co-localization channel is used to mask the original DAPI and CSFE channels, thus defining the single-positive DAPI^+^ and CSFE^+^ subsets. Using the HSB surface creation workflow, surfaces of any cell is created in a one step process, allowing for cell subsets to be defined during analysis in flow cytometry plots. Scale bar, 20 µm. Abbreviations: DAPI 4’,6-Diamidino-2-Phenylindole Dihydrochloride, CFSE carboxyfluorescein succinimidyl ester

Further differences in the practical application of the traditional method of surface creation and the HSB surface creation workflow are revealed with a confocal acquired two-dimensional three color image of splenocytes. The splenocytes have been labeled with different combinations of 4’,6-Diamidino-2-Phenylindole, Dihydrochloride (DAPI), carboxyfluorescein succinimidyl ester (CFSE), and propidium iodide to give single-, double-, and triple-positive cells. As the stains from the different dyes did not fully overlap, the surfaces for single cells created using the traditional method was often split or incomplete (Fig. 3a). However, the cells were rendered as complete surfaces with the HSB surface creation method because the signals from all markers were merged in the brightness channel. The HSB surface creation method is thus uniquely suited for quantitative measures of dyes that only partially stain the cell membrane, such as the CD62L antibody that binds only to neutrophil pseudopods^11^.

As previously mentioned, in the traditional workflow, cell subsets have to be defined early on in the process, during the creation of the co-localization channels. This implies that the cells are gated into their individual populations during the process of surface creation (Fig. 3b), eliminating the possibility of having a global view of the different cell populations. In contrast, when we attempted the HSB surface creation workflow, we were able to define the cell subsets after surface creation, using either statistics filters in Imaris (Supplementary Fig. 2), or in a histocytometry manner by the adjustments of population gates. This ability to gate the cells into their individual subsets later on in the workflow provided us with more flexibility and objectivity during analysis.

**Fig. 4:**
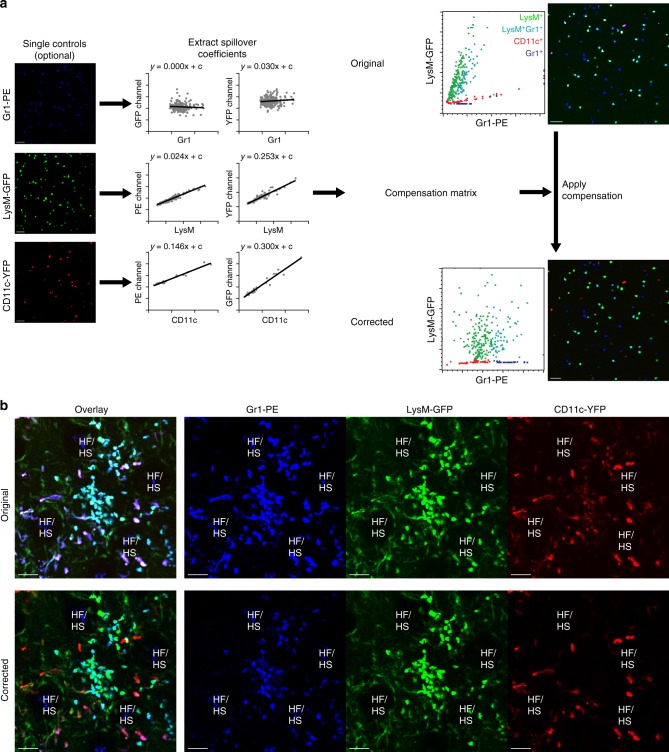
Spectral compensation using the HSB surface creation workflow. **a** Spectral compensation process. Spillover coefficients are extracted from the single stain controls and entered into a compensation matrix. Single stain controls may not be required if single-positive cells are available in the image. Values from the matrix can then be applied to the histocytometry plots as well as the original image to generate a corrected image. **b** Spectral compensation applied to the individual channels of a snapshot of an injured transgenic LysM GFP—CD11c YFP mouse ear injected with GR1-PE. Abbreviations: LysM GFP Lysozyme M green fluorescent protein, CD11c YFP CD11c yellow fluorescent protein, GR1-PE anti-granulocyte receptor-1 tagged with phycoerythrin, HF/HS hair follicle/hair shaft. Scale bar, 50 µm

Another critical advantage that the HSB surface creation workflow provides is the ability to carry out spectral compensation later on in the workflow during analysis. Within the traditional surface creation workflow, it is necessary for the user to first carry out channel arithmetic prior to the creation of co-localization channels, to ensure that only true co-localizing pixels are picked up in the channel. However, any error made during channel arithmetic requires the user to re-create the co-localization channels and carry out surface creation again (Supplementary Fig. 3a). With the HSB surface creation workflow, spectral compensation can be applied during data analysis (Supplementary Fig. 3b), thus if there is any error, it can be corrected without the need for re-generating surfaces. To do this, the user can extract spillover coefficients from the single stain controls to generate a compensation matrix. The compensation matrix can then be applied to generate a corrected image (Fig. 4a). In this three color image of an injured ear skin of Lysozyme M (LysM) green fluorescent protein (GFP) × CD11c yellow fluorescent protein (YFP) mouse that was injected with anti-granulocyte receptor-1 tagged with phycoerythrin (GR1-PE), the spillover from the individual channels can be sufficiently corrected such that only the neutrophils are seen in the GR1-PE channel, and the dendritic cells are primarily CD11c^+^, even when they were triple-positive in the uncorrected picture (Fig. 4b).

### HSB surface creation workflow in real-life imaging

**Fig. 5:**
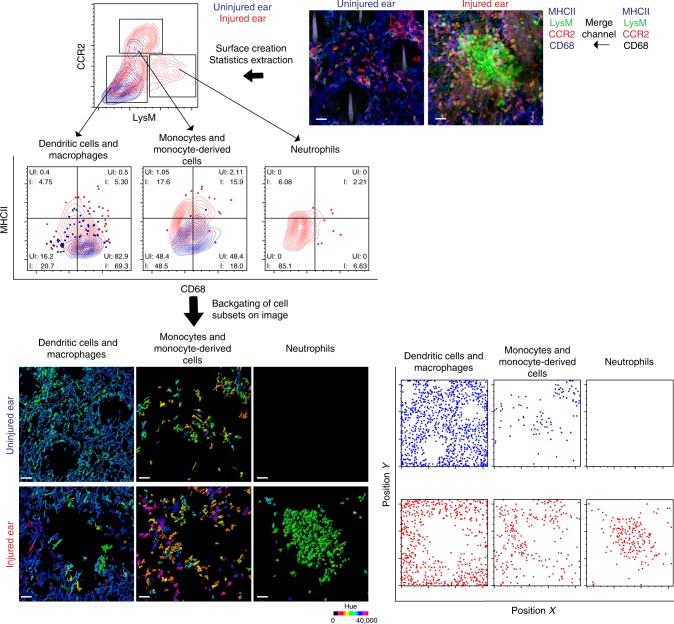
Application of HSB surface creation to whole-mount immunostaining. Application of the HSB surface creation workflow to a whole-mount immunostaining of a LysM GFP-CCR2 RFP mouse ear 180 min after injury with a needle stick. Mouse ear was immunostained with MHCII and CD68. The MHCII and CD68 channels were merged, followed by processing with the HSB surface creation workflow for surface creation and statistics extraction. Statistics from the cell surfaces were then exported to FlowJo for population gating. Gates for MHCII and CD68 were defined using the neutrophil population, and numbers in each quadrant represent the percentage of the total cell population existing in the respective quadrant. Cell subsets were then backgated onto the surfaces and rendered in their native hue. The relative X and Y positions of each subset are also demonstrated. Results are representative of two mice. Abbreviations: LysM GFP Lysozyme M green fluorescent protein, CCR2 RFP C-C chemokine receptor type 2 red fluorescent protein, MHCII major histocompatibility complex class II, UI uninjured, I injured. Scale bar, 30 µm

To illustrate the applicability of our approach in surfacing multicolor images, we examined the immunostained whole-mount ear skin of a needlestick injury applied to a LysM GFP × C-C chemokine receptor type 2 red fluorescent protein (CCR2 RFP) transgenic mouse, which was co-stained with major histocompatibility complex class II (MHCII) and the pan-macrophage marker CD68 (Fig. 5). Three main cell subsets—neutrophils, monocytes, and other myeloid cells (dendritic cells and macrophages)—could be identified by their expression levels of LysM and CCR2. In response to injury, neutrophils expressing high levels of LysM GFP infiltrated into the site of injury together with some MHCII^+^–CCR2^+^ monocytes and their derived cells, which upregulated MHCII expression (Fig. 5). However, MHCII^+^CCR2^−^ dendritic cells and macrophages remained in the periphery of injury site, but also displayed upregulation of MHCII. The cell subsets can be backgated onto the created surfaces for successful visualization of their spatial localization and morphology in the image.

**Fig. 6:**
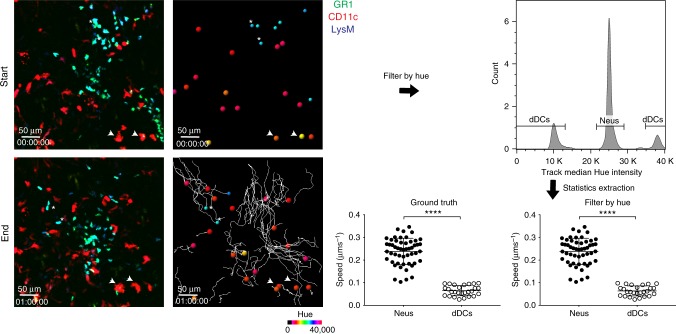
Application of HSB surface creation workflow to dynamic imaging. Application of HSB surface creation workflow to a dynamic image of a LysMGFP-CD11cYFP mouse injected with anti-GR1 antibody. The ear was injured with a needle stick, and LysM^+^ GR1^+^ neutrophils (asterisk) and CD11c^+^ or CD11c^+^ LysM^+^ dendritic cells (arrowhead) recruited to the injured region 30 min after the injury are tracked. Spots are rendered in the median hue of the cell. Tracks of the neutrophils and dendritic cells were gated based on the track median hue intensity, and the velocities of the different cell subsets plotted and compared to ground truth. Results are representative of two mice; ****p < 0.0001 using the two-tailed unpaired *t*-test. Difference in cell speed for ground truth dataset, *p* = 2.72 × 10^−24^, 95% confidence interval = 0.15–0.1965 µm s^−1^; difference in cell speed for test dataset, *p* = 6.14 × 10^−24^, 95% CI = 0.1509–0.1981 µm s^−1^. Abbreviations: LysM GFP Lysozyme M green fluorescent protein, CD11c YFP CD11c yellow fluorescent protein, GR-1 granulocyte receptor-1

Other than static images, the HSB surface creation workflow can also be applied to dynamic imaging. In this example of a ear needlestick injury in a LysM GFP × CD11c YFP transgenic mouse injected with GR-1 PE to stain for neutrophils, the tracks of the Lys M^+^–GR-1^+^ neutrophils can be accurately distinguished from either CD11c^+^ or LysM^+^–CD11c^+^ expressing dendritic cells, based on the average track hue median intensity (Fig. 6, Supplementary Movie 1). Manual tracking through visual identification of dendritic cells and neutrophils in each frame using the LysM and CD11c channels was used to generate ground truth tracks for each type of cell. These tracks were then compared to those filtered by hue, and the precision and recall rates to determine the accuracy of filtering were calculated. Precision rates (likelihood that tracks of neutrophils or dendritic cells were accurately classified) of 100% were obtained for both dendritic cells and neutrophils; while recall rates (probability that true positive tracks of neutrophils or dendritic cells are detected by filtering) of 96.06 and 100% were obtained for dendritic cells and neutrophils respectively (Supplementary Table 2). The HSB script can thus be applied in situations where auto-tracking of different cell types is carried out to easily filter cells of different hues for datasets acquired from dynamic imaging experiments.

## Discussion

The standard method of surface creation for multi-parametric images often involves a lengthy and tedious process that becomes a bottleneck especially when dealing with more than three different cellular markers, rendering analysis of such images difficult. We have demonstrated here the application of the HSB color space model to streamline the process of surface creation for carrying out histocytometry, which we have termed HSB surface creation. This workflow circumvents the need for the creation of co-localization channels, by generating a single brightness channel instead that can be used to create surfaces from multiple cell types at once. This step also solves the common problem of incompletely created surfaces due to partial co-localization of markers, which may be particularly important for the accuracy of obtaining relevant statistics, such as cell size and morphology.

With the HSB surface creation workflow, all cell surfaces are simultaneously created in the same step. Consequently, cell subsets can be defined much later with histocytometry, using population gating strategies similar to the style used with flow cytometry. This provides tremendous flexibility since it would be possible to correct any errors in the gating strategy without recreating the cell surfaces. It also provides the user with a global overview of all the cells before making decisions on the identity of the cell subsets. After feature extraction, cell subsetting can then be carried out in an objective or unbiased manner using a variety of machine-learning algorithms such as t-distributed stochastic neighbor embedding algorithm^12^ or *k*-means clustering^13^. Another powerful advantage of HSB surface creation is that it permits the user to carry out spectral compensation akin to flow cytometry data with the appropriate controls. Currently, compensation after surface creation is not possible in the traditional method as the spillover from one channel to another may cause single-positive cells to appear double-positive, necessitating the compulsory removal of any spillover signals very early in the workflow. Even with the appropriate controls, users working with flow cytometry often have to carry out tweaks to the spectral compensation matrix in order to get the cells to be correctly represented. With HSB surface creation, this is also as simple as changing a few numbers, but for the traditional workflow, this necessitates recreating the unmixed channels and redoing practically everything from scratch.

As with any method, the HSB surface creation workflow has its limitations. One key issue is that there can only be three channels input into the HSB conversion algorithm. This would be a problem if more than three markers were used in the image. To circumvent this problem, we have provided a script that generates the maximum intensity projection of multiple channels in one channel for surface creation. Appropriate channels can be chosen for input into the HSB conversion algorithm to generate hue and saturation channels that are biologically meaningful. The information for each channel can then be extracted and gated using histocytometry by loading in individual channels after surface creation (Supplementary Figs. 4 and 5). Note that we have already successfully employed this strategy in our example for Fig. 4. Strategic planning of the multiplex staining panel would also help in deciding which channels to convert to the HSB channels for surface creation. Most importantly, the user should only select relevant co-localizing markers to input in the algorithm.

Other minor considerations when deciding on the antibody panel for HSB surface creation include selecting at least one marker that will cover the entire cell (for each cell type of interest) in order to ensure the creation of complete surfaces. The user should also find a method for cell segmentation to ensure that the surfaces generated are accurate, as there is a high tendency for the cells to become tightly packed since all the cells will be rendered in the brightness channel. Strategic planning of the markers to render for surface creation would also minimize the possibility of too many touching cells.

In conclusion, the HSB surface creation workflow is designed to provide biologists with a method that can be applied to multiplex fluorescent microscopy images that is adaptable to their unique image analysis needs. HSB surface creation is applicable to different modalities of imaging—whether it is confocal, multi-photon, or light sheet microscopy, as well as different types of datasets, such as static whole-mount immunostains or dynamic images. The scripts for this method apply the standard equations that convert the channels in RGB to HSB with minor modifications (Supplementary Note 2). For the generalist biologist with minimal scripting experience, a manual and a step-by step tutorial explaining how to use each script is available as a reference (see section on Code Availability). Users familiar with programming can easily modify the code for their own applications as the scripts are written in an open source programming language Python. Alternatively, they can integrate the scripts into their workflows through the command line interface. We have also included some utility scripts to ensure that the users’ raw data files can be read by the HSB conversion script, and included a custom-made look up table for visualizing the hue of the cell for the convenience of the users in Fiji and Imaris. In summary, HSB surface creation can be easily integrated into the user’s image analysis workflow, and we envision that it will greatly improve the ease of quantitative analysis, enabling biologists to derive more out of their imaging data.

## Materials and methods

### Implementation of Python scripts

Hue-saturation-brightness surface creation scripts were written using Python 3.5, and tested for compatibility with WinPython 3.5.1.

### Mouse strains

All mice (*Mus musculus*) were bred and maintained under specific pathogen–free conditions in the Biological Resource Centre (BRC) of A*STAR, Singapore. Stock mice *Lyz2*^*gfp/gfp*^ (*Lyz2tm1.1Graf*), *Ccr2*^*rfp/rfp*^ (B6.129(Cg)-*Ccr2tm2.1Ifc/J*), and CD11c-YFP (Tg(Itgax-Venus)1Mnz) were used for breeding to generate *Lyz2*^*gfp/+*^*Ccr2*^*rfp/+*^ mice and Lyz2^*gfp*/+^ CD11c-YFP mice. Mice were maintained on a C57BL/6J background and all experiments were performed under the approval of the Institutional Animal Care and Use Committee (IACUC) of the BRC, in accordance with the guidelines of the Agri-Food and Veterinary Authority (AVA) and the National Advisory Committee for Laboratory Animal Research (NACLAR) of Singapore. Male and female mice between 6 and 13 weeks old were used for the experiments and no formal randomization of mice was done.

### Antibodies

Primary antibodies used: CD68 (clone FA-11, eBioscience, catalog no.: 14-0681-82); MHC Class II (I-A/I-E) Monoclonal Antibody, eFluor 450, (clone M5/114.15.2, eBioscience, catalog no.: 12-5321-82); phycoerythrin conjugated anti-mouse Ly6G/Ly6C (GR-1) (clone RB6-8C5, Biolegend, catalog no.: 108408); CD4 (Abcam, catalog no.: EPR19514); CD11b (Abcam, catalog no.: EPR1344); CD45R (clone RA3-6B2, Abcam, catalog no.: ab64100); S100A9 (clone 2B10, Abcam, catalog no.:ab105472); CD3e (Abcam, catalog no.: ab5690).

Secondary antibodies used: Alexa Fluor 488 anti-rat antibody (Life Technologies, catalog no: A21208); Alexa Fluor 647 anti-rat antibody (Life Technologies, catalog no.: A2124); Alexa Fluor 647 anti-rabbit antibody (Life Technologies, catalog no.: A31573).

### In vitro staining of splenocytes

Splenocytes were isolated from the spleens of wild-type C57BL/6J mice by mashing. Fixed cells were labeled with 7.5 μM DAPI, 5 μM of CFSE, and 1.5 nM of propidium iodide at room temperature for 15 min in a manner that ensured that there were cells that were only positive for each of the dyes, cells that were positive for two out of the three dyes and cells that were stained with all three types of dyes. Splenocytes were washed with PBS after staining.

### Needle stick injury model

Mice were anaesthetized with an intra-peritoneal injection of ketamine-xylazine (15 mg mL^−1^ ketamine and 1 mg mL^−1^ xylazine dissolved in sterile water; 8 μL g^−1^ bodyweight). Ears were depilated using the Veet hair removal cream (Reckitt Benckiser), and placed on a custom-built platform with regulated heating for the mouse body (36.5 °C) and mouse ear (35 °C). 5 min after the injection, the ear skin was jabbed once using an insulin syringe fitted with a 31G needle (Becton Dickinson), in close proximity to the macroscopically visible post-capillary venules and arterioles.

### Immunostaining procedure

Mouse ears were collected at 2 h post injury and fixed for 12 h at 4 °C in 2% paraformaldehyde with 30% sucrose. Ears were then washed, split and decartilaged before they were blocked overnight in staining buffer consisting of PBS with 0.3% Triton X-100, 0.2% bovine serum albumin, 2.5% goat serum and 2.5% donkey serum with 5% DMSO. Ears were then incubated with primary antibody against CD68 (clone FA-11, eBioscience) using a 1:200 dilution for 1 day, washed and incubated with Alexa Fluor 647 anti-rat antibody (Life Technologies) using a 1:500 dilution for 1 day, followed by washing and incubation with eFluor 450 MHCII (M5/114.15.2, eBioscience) using a 1:100 dilution for 1 day. Ears were then washed and optically cleared. The experiment was carried out once.

### Optical clearing

Immunostained ears were optically cleared by incubation for 15 min with a proprietary refractive index matching medium RapidClear-Clarity Specific (RC-CS) solution (Refractive Index = 1.47) (SunJin Labs) at room temperature before they were mounted for image acquisition on the confocal microscope.

### Confocal image acquisition

Images of splenocytes and mouse ear were acquired using the Olympus inverted FV1000 confocal microscope, using the Olympus UPLSAPO ×40 objective with a numerical aperture (N.A.) of 0.95. Fluorophores were excited with the 405, 488, 535, and 635 nm laser lines.

### Mouse ear whole-mount image processing

Background pixels in the image were removed by setting thresholds for every 10 Z slices. The images underwent log transformation followed by mean filtering using a radius of 2.0 pixels. The channels for CD68 and MHCII were merged into a single channel using a script picking pixels with maximum intensity from either channel. The preprocessed channels were then transformed into the hue-saturation-brightness channels for surface creation.

### Cell surface creation

Cell surface creation was carried out using the Imaris 8.4 software (Bitplane), and surfacing was carried out in the channels they were redistributed in and manually inspected for accuracy.

### Statistics extraction and FCS conversion

The original channels, logarithmic transformed channels and HSB transformed channels were loaded into the Imaris file with cell surfaces, and the parameters of interest were then saved out as a comma separated value file. Comma separated value files were then dragged into the FlowJo v.10.1 (Treestar Inc.) workspace where it is automatically converted into a flow cytometry standard format file.

### Visualization of surfaces in Imaris

Surfaced cells were gated in FlowJo, and saved out as a comma separated value file. The cell IDs were used to visualize the surfaces in the surfaced Imaris file.

### Intravital multiphoton imaging

Prior to imaging, 2 µg of anti-granulocyte receptor-1 phycoerythrin was injected into the mouse intravenously. Mouse ear skin was imaged using LaVision TriM Scope II microscope (LaVision BioTec), using a ×20 NA 1.4, water immersion objective lens and a Coherent Chameleon pulsed infrared laser. An excitation wavelength of 990 nm was used to excite GFP, YFP, phycoerythrin, and second harmonic generation simultaneously. Emission wavelength was collected using 500/24, 531/22, 579/34, and 494/41 nm filters, respectively. To acquire time-lapsed data, the 60 μm-thick stack (step size; 4 μm) was acquired every 30 s for 1 h, conducted 30 min after the needlestick injury. Using the Imaris software, channel arithmetic was applied to correct for channel spillover. The time-lapse images were then transformed into the hue-saturation-brightness channels for rendering. Experiment was replicated at least twice.

### Post-processing of time-lapse imaging

Dermal dendritic cells and neutrophils were tracked using the YFP and GFP channels respectively in the Imaris software (Bitplane). These tracks were treated as ground truth. The tracks were then merged, and filtered by the track intensity median hue using appropriate hue values for dermal dendritic cells and neutrophils. Two-tailed unpaired Student’s *t*-test was used to determine the difference in speed between dermal dendritic cells and neutrophils. Precision and recall rates for each cell type were then calculated for each time-lapse imaging experiment (*n* = 2).

### Spectral compensation

Bone marrow cells from LysM GFP and C57BL/6J mice, as well as splenocytes from CD11c YFP mice were used for spillover correction. Single stains of LysM GFP, CD11c YFP cells, and GR1-PE stained cells were imaged using the multiphoton microscope. An excitation wavelength of 990 nm was used to excite GFP, YFP, and PE simultaneously. Emission wavelength was collected using 500/24, 531/22, 579/34, and 494/41 nm filters, respectively. Images were corrected based on the compensation matrix derived from the single stains, and spillover coefficients were derived by extracting channel statistics for the single stains and plotting the linear correlations in Prism version 7 (Graphpad).

### Sequential immunofluorescence staining

A total of 4 µm formalin fixed paraffin embedded spleen sections from C57BL/6J mice were used for immunofluorescence staining. Sections were rehydrated in an ethanol gradient, following which heat mediated antigen retrieval was carried out at 95 °C and slides were cooled to room temperature by immersion in tap water. Slides were then blocked with a PBS buffer containing 5% donkey serum, 2.5% bovine serum albumin, and 0.1% Tween-20, following which, primary antibodies at a 1:100 dilution in the blocking buffer were added for incubation at room temperature. Excess primary antibody was then washed off and slides were incubated with the appropriate secondary antibodies in the green and far-red channels using a 1:500 dilution. Cell nuclei were then stained using propidium iodide (Sigma-Aldrich) before images were acquired on the Evos FL Imaging system (Thermo Fisher). Antibodies were stripped by 30 min incubation at 50 °C in glycine-SDS buffer (pH 2) buffer, following which protein blocking and blocking of any remaining primary antibody was carried out using the appropriate Alexa Fluor 405 conjugated secondary antibodies using a 1:100 dilution. The next round of antibodies for immunofluorescence staining was then added. Images were stitched using the Microscope Image Stitching Plugin (MIST)^14^, while images from multiple rounds of imaging were registered using the nuclei channel with the aid of the Register Virtual Stack plugin from FIJI^15^. Experiment was carried out once.

The primary antibodies used were: anti-S100A9 (Clone 2B10, Abcam), CD4 (Abcam), CD3 (Abcam), B220 (Clone RA3-6B2, Abcam), CD11b (Abcam), and F4/80 (Clone CI:A3-1, Biolegend). The secondary antibodies used were: Alexa Fluor 488 anti-rat antibody (Life Technologies); Alexa Fluor 647 anti-rat antibody (Life Technologies); Alexa Fluor 647 anti-rabbit antibody (Life Technologies).

### Software

The following software was used for analysis and processing of the data presented in this paper: Custom-written code for WinPython 3.5.1 (Hue-saturation-brightness surface creation) is made available together with the manuscript; Graphpad Prism (version 7) for statistical analysis; FlowJo (version 10.1) for flow-cytometry style analysis; Imaris (version 8.4); FIJI (version 1.50e) for image analysis and Adobe Illustrator (Creative Suite 6) to make figures.

### Code availability

The codes for the HSB surface creation workflow, test dataset, and the manual and step-by-step tutorial detailing the steps for using the code are available in GitHub (https://github.com/HSBsurfacecreation/Hue-Saturation-Brightness-based-surface-creation). The codes are licensed under the MIT license.

## Electronic supplementary material

Supplementary Information

Description of additional supplementary items

Supplementary Movie 1

## Data Availability

The data that support the findings of this study are available from the corresponding author upon reasonable request. The associated Github DOI is: 10.5281/zenodo.1311586^16^.
