# Supplementary material for: Streamlining volumetric multi-channel image cytometry using hue-saturation-brightness-based surface creation
Source: Commun Biol. 2018 Sep 6;1:136. doi: 10.1038/s42003-018-0139-y (PMC6127105; doi:10.1038/s42003-018-0139-y)
Supplement: Supplementary file 1 — Supplementary Information [file 42003_2018_139_MOESM1_ESM.pdf]

Supplementary Figures

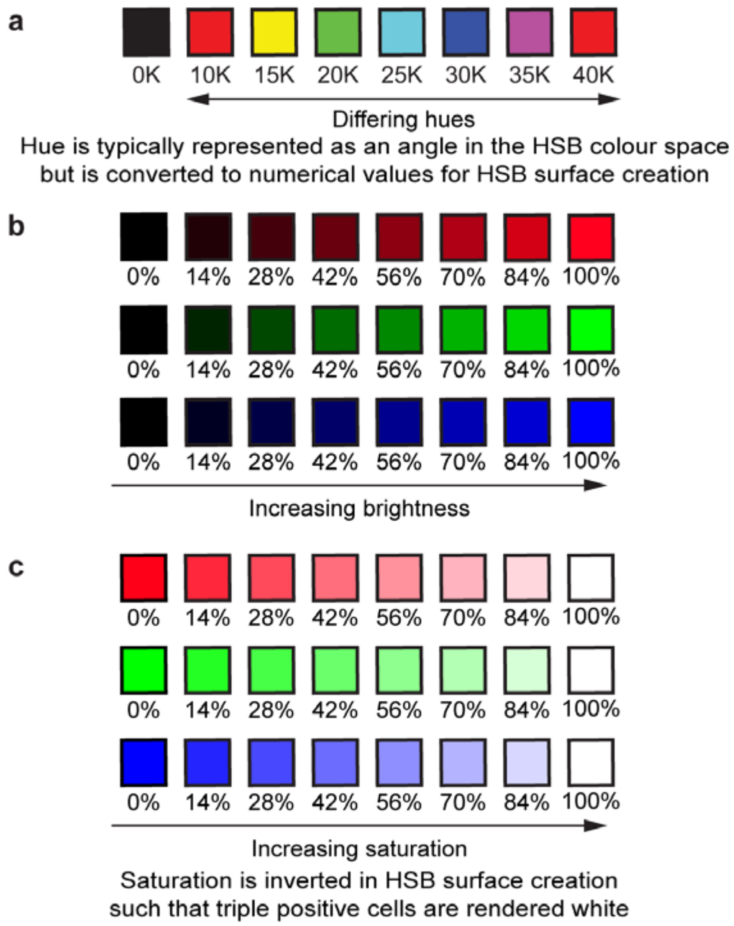

Supplementary Figure 1 Representations of individual components of the HSB colour model.

(a) Linear numerical representation of hues in the HSB surface creation algorithm. Red is represented by 10 000 and 40 000, green is represented by 20 000 and blue is represented by 30 000.

(b) 1 dimensional representation of the brightness channel demonstrating increasing intensities for red, green and blue colours.

(c) 1 dimensional representation of the saturation channel demonstrating increasing saturation for red, green and blue colours.

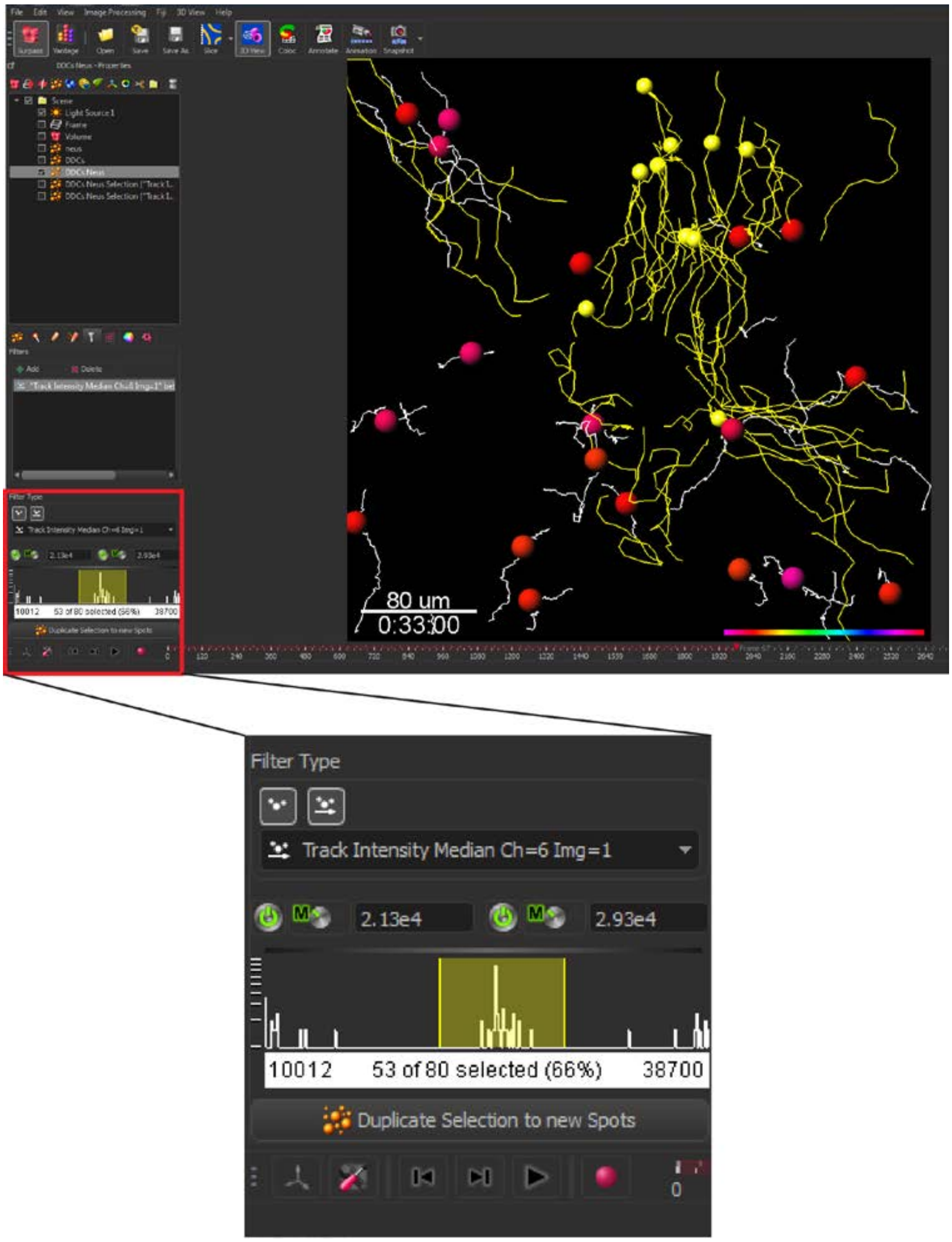

Supplementary Figure 2 Screenshot showing cell subsetting using statistics filters in Imaris.
One way of defining cell subsets is to use the statistics filters in Imaris. Zoomed in view of tracks selected using track intensity median hue values between 2.13 x 10^4 and 2.93 x 10^4. Selected cells tracks are highlighted in yellow.

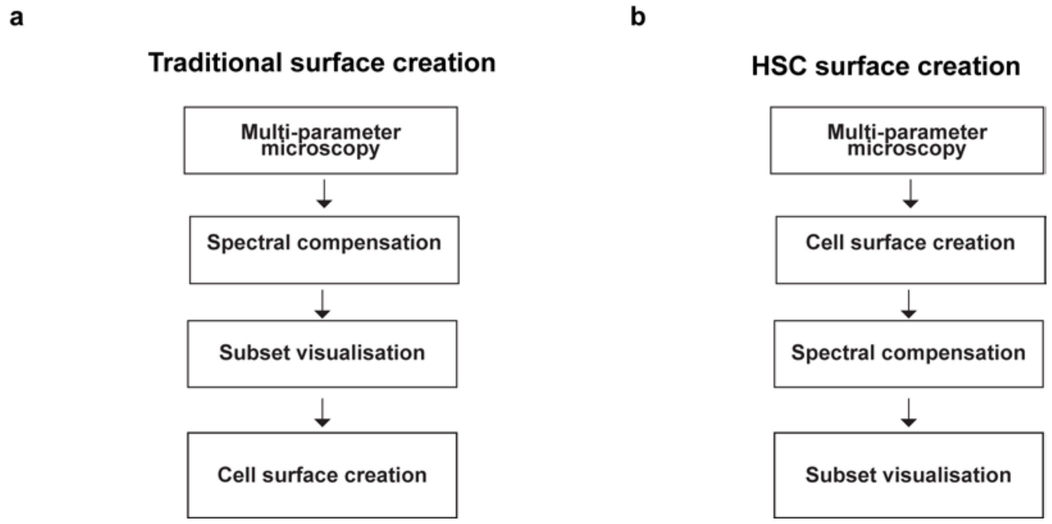

Supplementary Figure 3 Spectral compensation using traditional surface creation and HSB surface creation workflows.

(a) Spectral compensation using traditional surface creation. Images are acquired, and the raw images are corrected based on the spillover coefficients derived from the single stains. Cell subsets are then visualized during the creation of co-localization channels, followed by surface creation.

(b) Spectral compensation using HSB surface creation. Images are acquired, and the information from all the channels merged into the brightness channel. Surfaces are then created based on the brightness channel, and the statistics extracted. Spectral compensation can then be carried out based on the spillover coefficients derived from the single stains. The cell subsets can then be visualized through analysis.

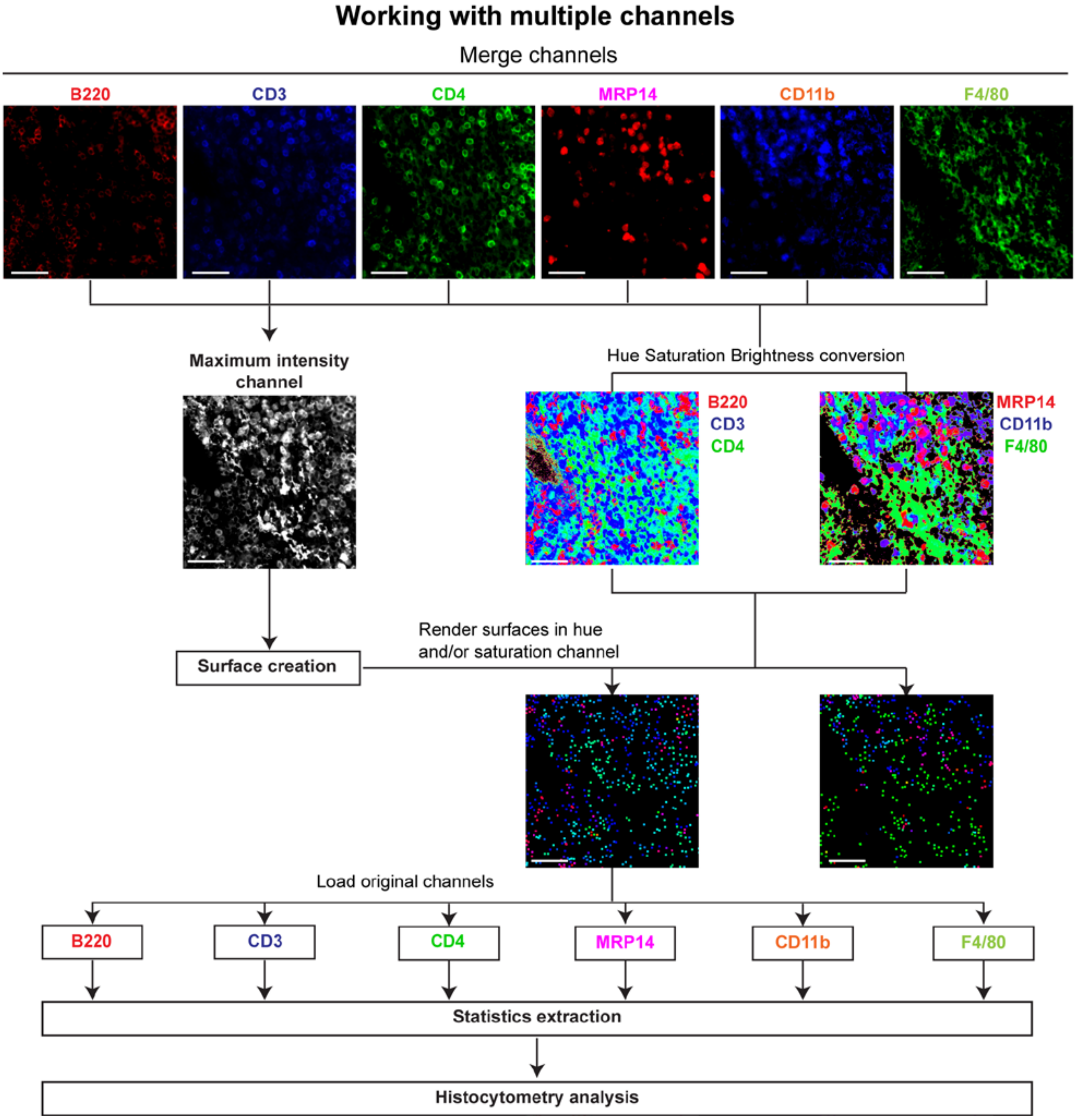

Supplementary Figure 4 Application of HSB surface creation to more than three channels. This example of a spleen section stained with 6 markers illustrates how HSB surface creation can be used for an image with more than 3 channels. The channels can be combined into a maximum intensity channel for surface creation, and specific channel combinations can be used for the hue-saturation-brightness conversion. Created surfaces are then rendered in the hue channel to check that the segmentation is accurate. The original channels can then be loaded for statistics extraction from the cell surfaces which can then be exported for histocytometry analysis or objective clustering using tSNE or *k*-means clustering. For the purposes of more accurate segmentation, we recommend that users include at least one cytoplasmic marker in their panel. Experiment was carried out once.

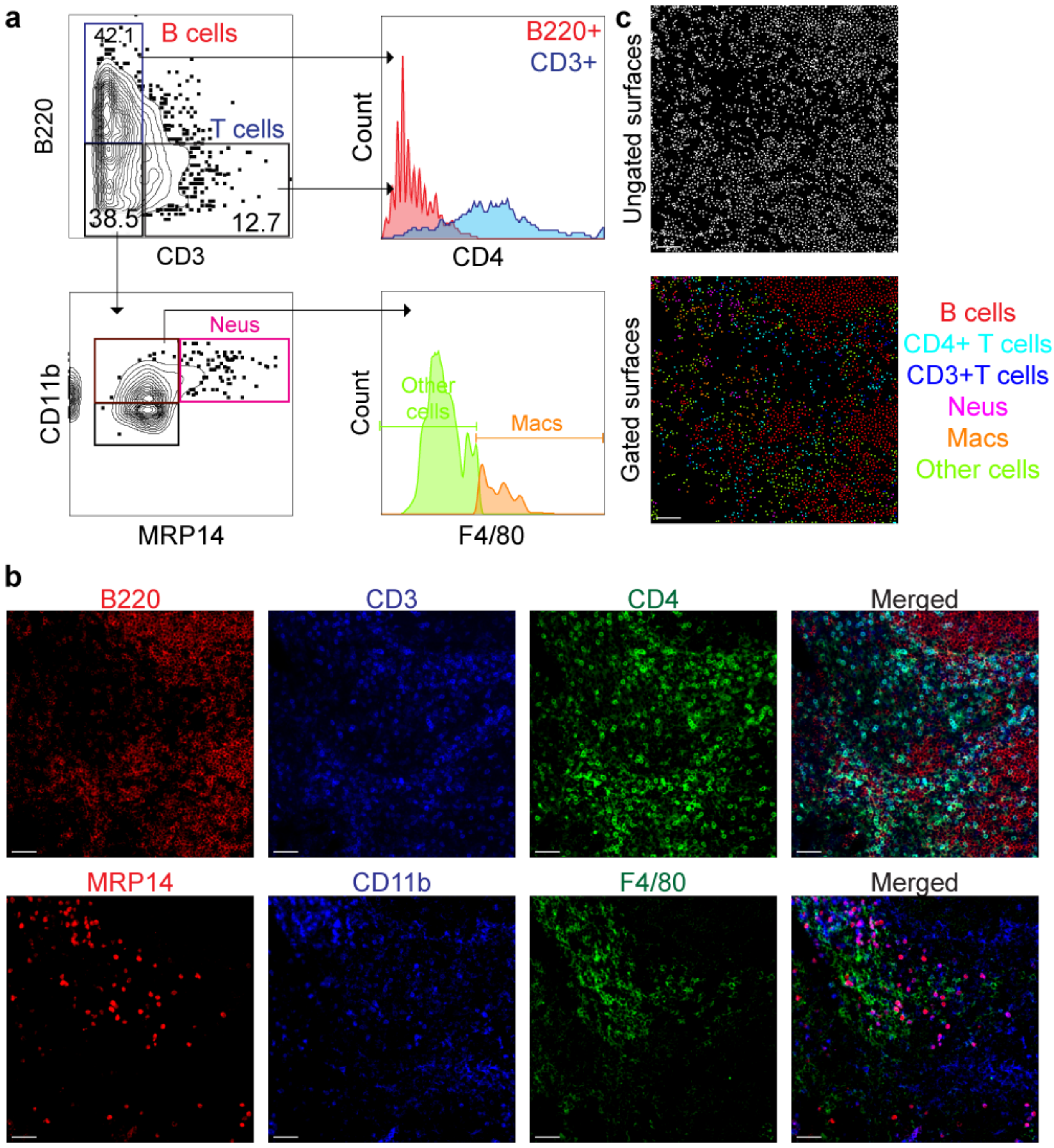

Supplementary Figure 5 Histocytometry analysis of spleen populations.
Illustration of histocytometry analysis of cell surfaces from spleen sections in Supplementary Figure 3.
(a) Surfaces were first gated on B220 and CD3, to derive T cell (CD3+), B cell (B220+) and double negative CD3-B220- populations. CD3+ T cells were further gated into CD3+CD4+ populations and CD3+CD4- populations using the B220+ population to define the CD4- population (not shown). CD3-B220- populations were then gated on MRP14 and CD11b expression to isolate the neutrophil (MRP14+), macrophage (CD11b+F4/80+) and other cell (CD11b+F4/80-) populations. Neus, neutrophils; macs, macrophages. Gates were drawn using thresholds of each individual channel in the image as a reference.
(b) Images of individual channels in the spleen section and the merged channels (CD3, CD4, B220, top; MRP14, F4/80, CD11b, bottom). Scale bar represents 200 µm.
(c) The isolated populations were then backgated onto the surfaces to identify the positions of the cell populations. Blue box, zoomed in image of B cells (B220+, red), CD4+ T cells (CD3+CD4+, cyan), CD3+ T cells (CD3+CD4-, blue), neutrophils (MRP14+, magenta) macrophages (CD11b+F4/80+, orange), other cells (CD11b+F4/80-, lime-green) populations. Scale bar represents 200 µm.

Number of cellular markers for multiplex staining	Number of possible cell subsets
1	1
2	3
3	7
4	15
5	31
6	63

Supplementary Table 1 Number of possible cell subsets depending on the number of cellular markers.

For an image *C* with the number of cellular markers *n*, where *r* is the number of markers that are multiplexed *C*(*n*,*r*), the number of possible cell subsets is expressed by the mathematical formula : ${}^{n}C_1 + {}^{n}C_2 + {}^{n}C_3 + \ldots + {}^{n}C_{r-1} + {}^{n}C_r$ or 2^n-1.

	**dDCs**	**Neus**
**Precision**	100 %	100 %
**Recall**	96.06 %	100 %

Supplementary Table 2 Precision & recall rates for dermal dendritic cells (dDCs) and neutrophils (Neus) after filtering by track median hue intensity.

Precision & recall rates for two representative time-lapse imaging experiments of dDCs and Neus responding to a needlestick injury were calculated and averaged. Precision was calculated in the following manner: true positives/ (true positives + false positives). Recall was calculated in the following manner: true positives/ (true positives + false negatives).

Supplementary Note 1 Application of customized look-up table (LUT) for hue rendering

Description of Look-up Table (LUT)

A look-up table is used for transforming input data into a certain format of colours for output.

In the HSC workflow, we have generated a customized look-up table that can be loaded into FIJI or Imaris software for rendering hue (Supplementary Figure 2a). The look-up table is designed such that background is assigned with the value of 0, and no pixels are assigned with the value of 1 to 9999. This avoids the problem of having a red hue for the background, making it easier for the user to visualize the hue of the actual cells.

Supplementary Note 2 Equations for conversion of image from Red-Green-Blue (RGB) colour model to Hue-Saturation-Brightness surface creation workflow

The Red-Green-Blue values are scaled by the dynamic ranges set by the user (i.e. maximum and minimum values are set by the user) to change the range to (0, 1) for each of the channels.

$$R' = \frac{R - Rmin}{Rmax - Rmin}$$

$$G' = \frac{G - Gmin}{Gmax - Gmin}$$

$$B' = \frac{B - Bmin}{Bmax - Bmin}$$

$M = (R', G', B')max$, where M represents the maximum RGB value of the image.

$m = (R', G', B')min$, where m represents the minimum RGB value of the image.

$C = M - m$, where C represents the chroma.

For hue calculation:

$H = 0$, where C = 0.
$H = \left(\left[\frac{G-B}{C}, \text{mod } 6\right] + 2\right) \times 5000$, where $M = R$
$H = \left([\frac{B-R}{C} + 4]\right) \times 5000$, where $M = G$
$H = \left([\frac{R-G}{C} + 6]\right) \times 5000$, where $M = B$

For brightness calculation:

$B = M \times 10\,000$

For saturation calculation:

$S = (1 - \frac{C}{B}) \times 10\,000$

The saturation calculation is inverted in the HSB surface creation workflow (as compared to traditional HSB models) to ensure that the colour white is rendered as a positive value, while background values will have a very low value.
