# Supplementary material for: Streamlining volumetric multi-channel image cytometry using hue-saturation-brightness-based surface creation
Source: Commun Biol. 2018 Sep 6;1:136. doi: 10.1038/s42003-018-0139-y (PMC6127105; doi:10.1038/s42003-018-0139-y)
Supplement: Supplementary file 2 — Description of additional supplementary items [file 42003_2018_139_MOESM2_ESM.docx]

**Description of additional supplementary items**

**Supplementary Movie 1 Dermal dendritic cells (dDCs) and neutrophil recruitment to a needlestick injury**

A time-lapse sequence of maximum projection showing the migratory pattern of dDCs and neutrophils in response to a needlestick injury. Tracked cells (left) are represented by spheres (right) coloured in the median hue of the cell, dDCs are generally represented as magenta or red; neutrophils are generally represented as green or cyan. The colour bar on the right represents the median hue intensity of the cell.
